# Supplementary material for: Site-specific ubiquitylation acts as a regulator of linker histone H1
Source: Nat Commun. 2021 Jun 9;12:3497. doi: 10.1038/s41467-021-23636-5 (PMC8190259; doi:10.1038/s41467-021-23636-5)
Supplement: Supplementary file 1 — Supplementary Information [file 41467_2021_23636_MOESM1_ESM.pdf]

Site-specific ubiquitylation acts as a regulator of linker histone H1

Höllmüller *et al.*

Supplementary Information

Supplementary Figures

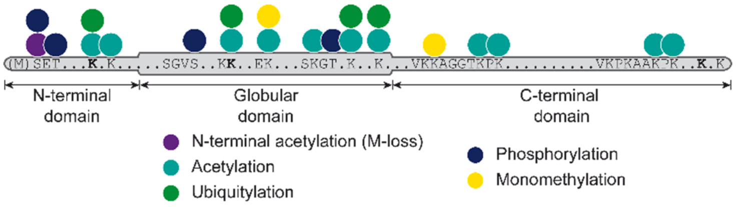

Supplementary Fig. 1: Endogenous modification sites of H1.2.
Schematic depiction of modification sites in H1.2 after IP-enrichment from HEK 293T cells and identification by LC-MS/MS. Positions investigated within this study are indicated in bold (K17, K64 and K206). Probably due to the high lysine content (40%) of the CTD resulting in relatively low sequence coverage, no ubiquitylation site was detected in the CTD of H1.2.

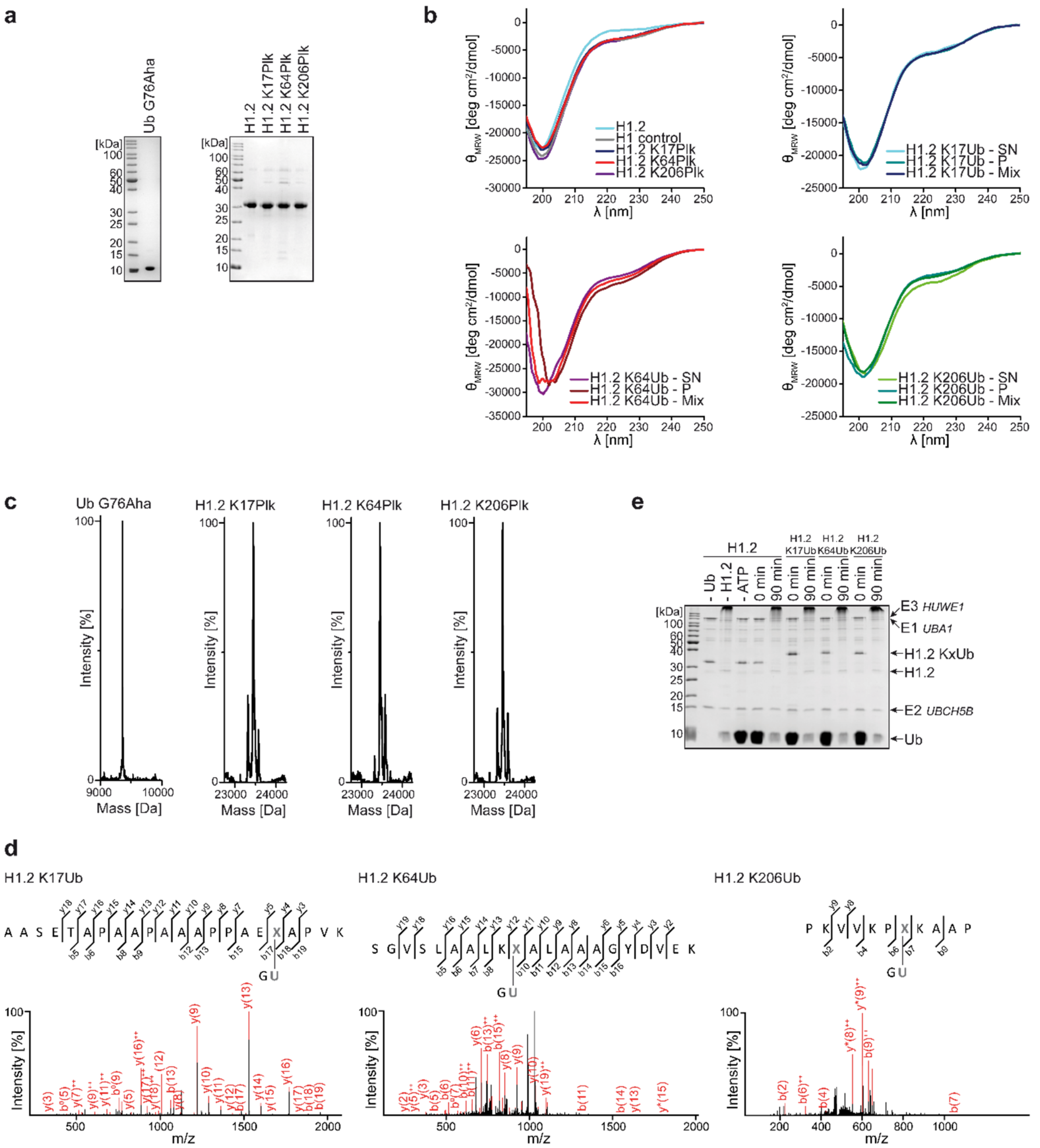

Protein	Peptide sequence	Precursor m/z [Da]	Charge	Mr(ex)	Mr(calc)	ΔM [Da]
H1.2 K17Ub	AASETAPAAAPAAAPPAE**X**(GU)APVK	1150.0885	2+	2298.1625	2298.1604	0.0021
H1.2 K64Ub	SGVSLAALK**X**(GU)ALAAAGYDVEK	782.4219	3+	2344.2440	2344.2386	0.0054
H1.2 K206Ub	PKVVKP**X**(GU)KAAP	482.6065	3+	1444.7977	1444.8514	-0.0537

Supplementary Fig. 2: Characterization of ubiquitylated histones.
a Ub G76Aha, H1.2 and H1.2 KxPlk variants after SDS-PAGE and Coomassie blue staining. **b** CD spectra of recombinant H1.2, H1.2 KxPlk variants and control H1 (Calbiochem) indicating protein refold after purification using denaturing conditions (top, left). CD spectra of ubiquitylated histones after click reaction and purification (top, right and bottom). SN = protein fraction obtained from the supernatant after click reaction, P = protein fraction obtained from pellet after click reaction, Mix = mixture of SN- and P-fractions (1:1). **c** LC-MS analysis of full-length proteins. Ub G76Aha calc. 9361 Da, measured 9360 Da; H1.2 K17Plk calc. 23452 Da, measured 23452 Da; H1.2 K64Plk calc. 23452 Da, measured 23452 Da; H1.2 K206Plk calc. 23452 Da, measured 23453 Da. **d** Representative spectra after LC-MS/MS analysis of ubiquitylated histones showing incorporation of the unnatural amino acids (X = Plk, U = Aha) at the respective sites and linkage *via* click reaction. **e** Ubiquitylation assay using H1.2 KxUbs from click reaction as substrate proteins.

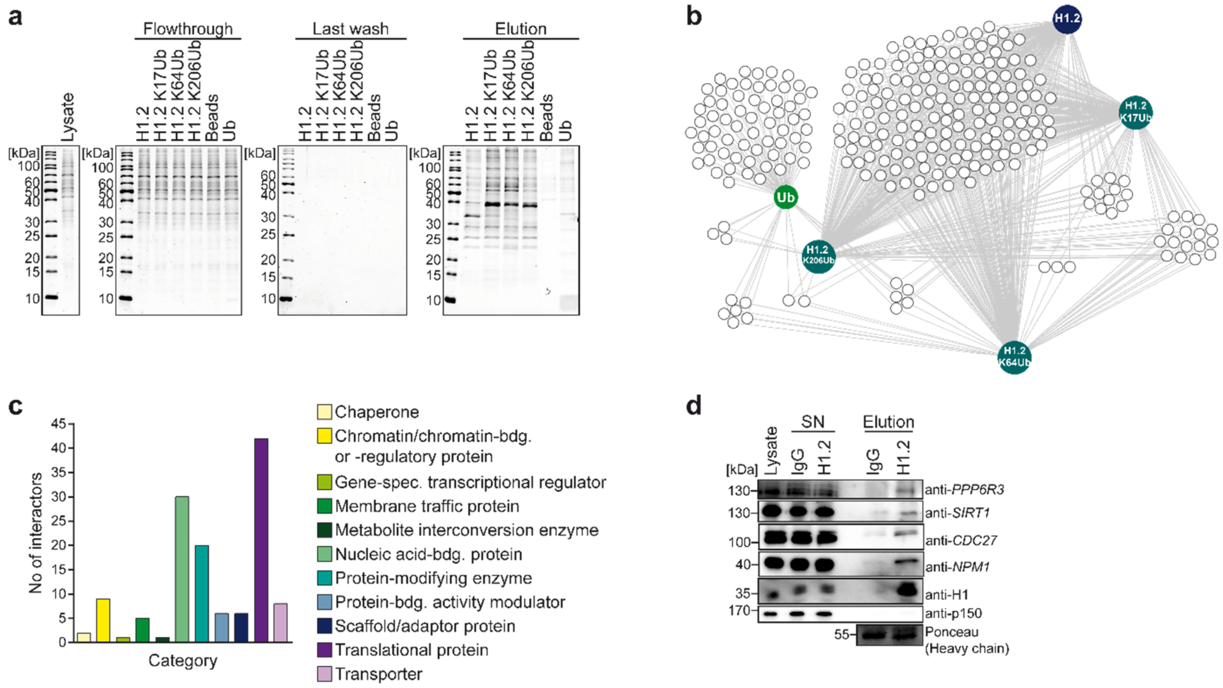

Supplementary Fig. 3: Identification of the H1.2 KxUb interactome by AP-MS.

a Analysis of the AP-MS assay by SDS-PAGE and Krypton protein staining. HEK 293T cell lysate was incubated with the respective bait proteins and Strep-Tactin beads. Flowthrough was collected and beads were washed five times. Bound proteins were eluted with desthiobiotin. **b** Schematic representation of identified interactions. Blue and green nodes represent the bait proteins H1.2, H1.2 KxUbs and Ub, white circles represent interactors, grey lines indicate protein-protein interactions. Most of the histone interacting proteins are found in the nucleus since also H1.2 is predominantly located in the nucleus. Non-nuclear interactors can be explained by the fact that H1, and especially H1.2, can also be translocated between nucleus and cytoplasm during the cell cycle[1,2] and as apoptotic signal following DNA double-strand breaks[3]. **c** GO-term analysis of all proteins binding to H1.2 KxUbs based on PANTHER 'Protein Classes'. **d** Co-immunoprecipitation analysis of interactors of endogenous H1.2 visualized by western blot analysis. Lysate indicates HEK 293T cell lysate as input, IgG indicates the control with an unspecific IgG antibody and H1.2 experiments with the anti-H1.2 antibody. SN marks supernatant.

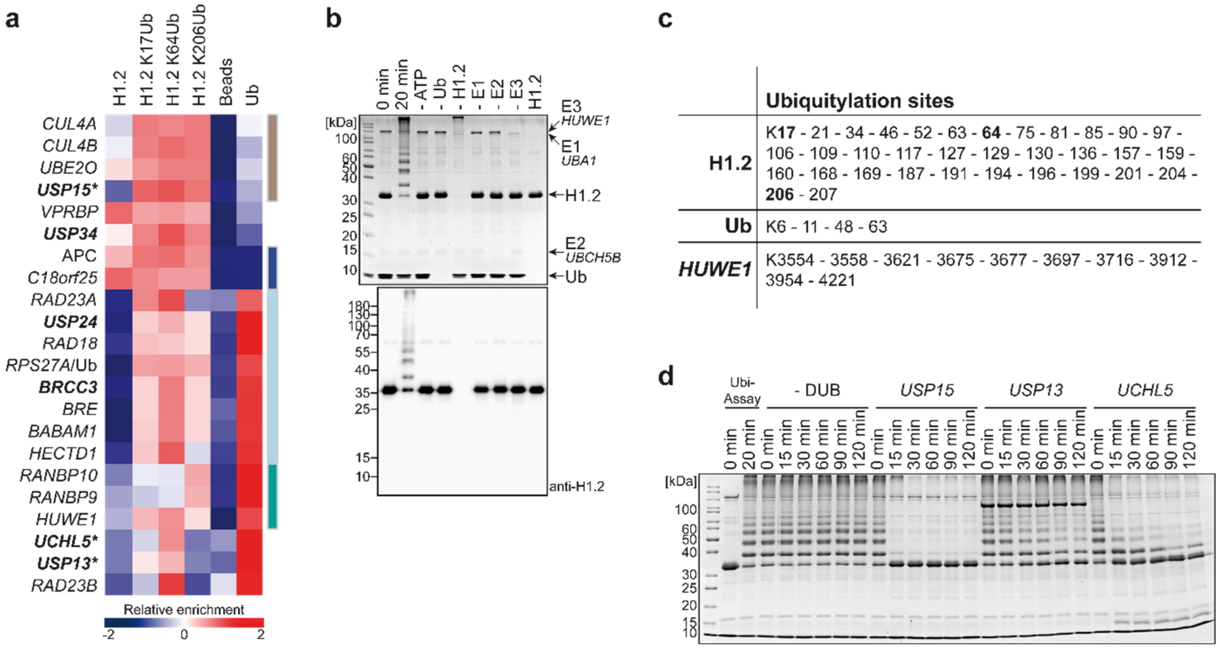

Supplementary Fig. 4: Characterization of DUBs and ubiquitylation-associated proteins identified by AP-MS.

a Heatmap indicating relative enrichment of proteins involved in protein ubiquitylation, deubiquitylation and Ub-binding pathways. APC represents the mean value of all subunits of the anaphase promoting complex/cyclosome detected as hits including *CDC27* isoform CRA_c and the adapter protein *FZR1*. DUBs are marked in bold; asterisks indicate DUBs used in deubiquitylation assays. **b** Ubiquitylation assay of H1.2. SDS-PAGE and Coomassie blue staining (top) and western blot analysis (bottom). The minus sign indicates that the respective component was not present in the reaction mixture. **c** Ubiquitylation sites generated by *in vitro* ubiquitylation assay with H1.2 as substrate protein. *HUWE1* = catalytic domain (1147 aa/aa 3228-4374). **d** Ubiquitylation and time-dependent deubiquitylation of H1.2. – DUB indicates that no DUB was added after the ubiquitylation assay and during subsequent incubation.

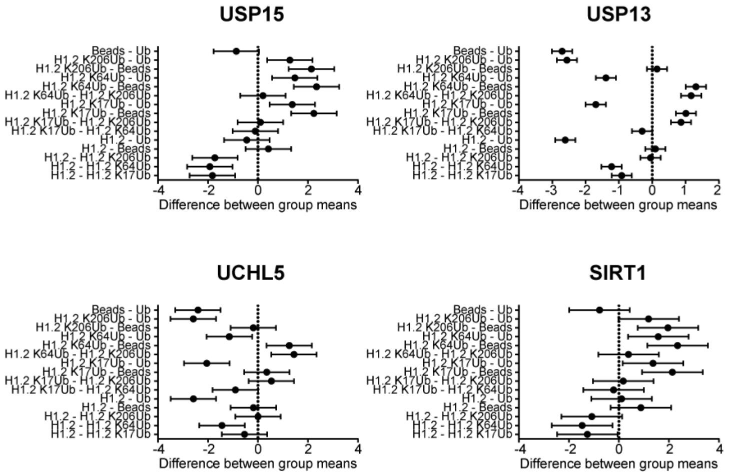

Supplementary Fig. 5: Results of Tukey's multiple comparisons test for selected interactors of H1.2 KxUb variants.

Diagrams show 95% CI of differences between group means, indicating significant differences after pairwise comparison of indicated samples after one-way ANOVA analysis (repeated measures) with Tukey's multiple comparisons test with α = 0.05, 95% confidence interval. Corresponding enrichment patterns are shown in Fig. 3a and c.

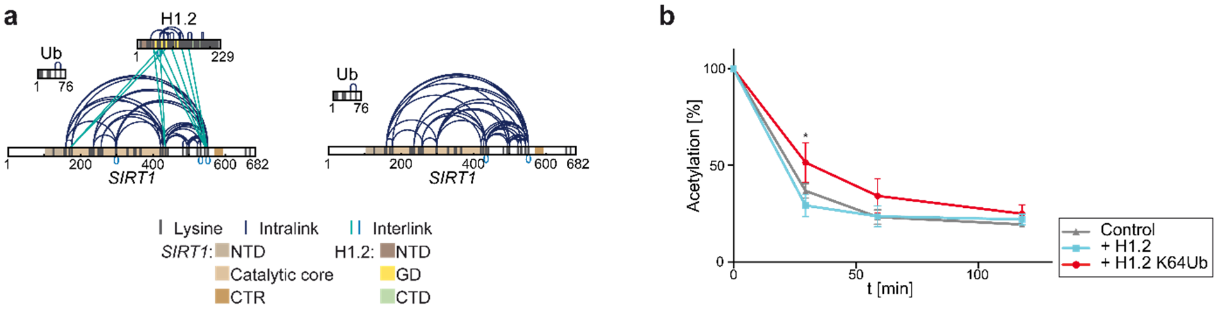

Supplementary Fig. 6: Characterization of interactions of H1.2 and H1.2 K64Ub with deacetylase *SIRT1*.

a Interactions of H1.2, Ub and *SIRT1* (left) as well as Ub and *SIRT1* (right) as identified by XL-MS. **b** *In vitro* deacetylation of substrate protein p53 K370AcK with *SIRT1* in the presence of H1.2, H1.2 K64Ub or in the absence of any histone (Control). The normalized intensity of p53-acetylation was plotted over time (for western blots see Fig. 3f). Data are presented as mean values +/- standard error of the mean, n = 3 independent experiments, two-way ANOVA with Tukey's multiple comparisons test with $\alpha = 0.05$, 95% confidence interval. * $0.01 < p \leq 0.05$ indicating the significant difference of measurements between samples (+ H1.2) and (+ H1.2 K64Ub) at t = 30 min with p = 0.0120.

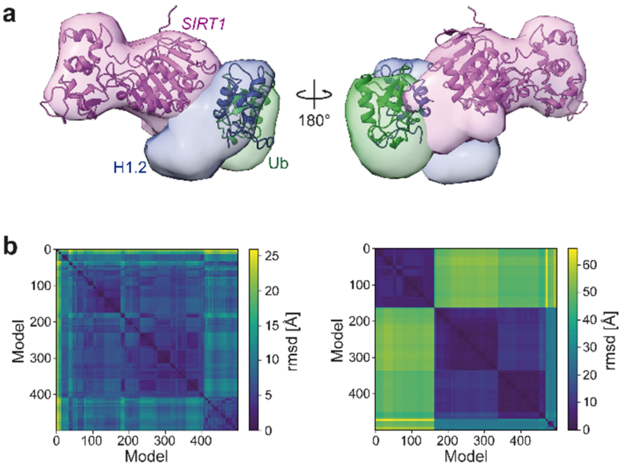

Supplementary Fig. 7: Structural modeling of H1.2: *SIRT1* and ubiquitylated H1.2: *SIRT1* complex.

a Bayesian crosslinking guided integrative structural modeling using the crystal structures of *SIRT1* (PDB: 4ig9), Ub (PDB: 1ubq) and the chicken H1 GD (PDB: 1ghc) together with our crosslinking data as input. Shown is the second main cluster of structural solutions for the H1.2 K64Ub: *SIRT1* complex. See Fig. 3e for the other main cluster. **b** Rmsd matrices of the 500 best-scoring models of the pooled sampling runs of H1.2: *SIRT1* (left) and H1.2 K64Ub: *SIRT1* (right). The run without Ub converged into a single cluster (see Fig. 3e), while the run with Ub converged into two main clusters. The bigger one of these two clusters can further be split into two sub-clusters which only differ in their in-place rotation. The bigger cluster is shown in Fig. 3e while the smaller cluster is shown in a (this Figure).

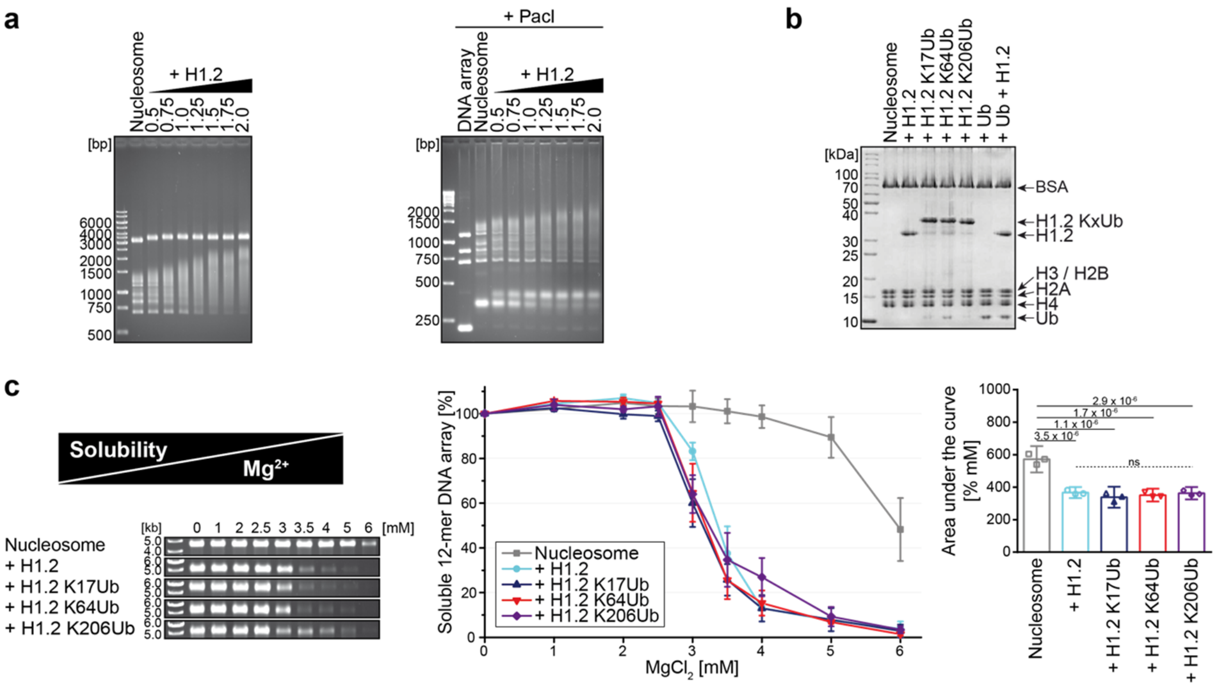

Supplementary Fig. 8: Analysis of chromatosome arrays assembled with H1.2 KxUbs.
a Chromatosome arrays were assembled with increasing concentrations of H1.2 and an EMSA was performed using agarose gels (left). For the analysis of H1.2-binding to the nucleosome, arrays were digested by PacI and analyzed by agarose gel electrophoresis (right). **b** Nucleosome and chromatosome arrays were analyzed by SDS-PAGE and Coomassie blue staining. **c** $MgCl_2$ precipitation assay for the analysis of the array compaction state (left). Arrays were incubated with increasing concentrations of $MgCl_2$. After centrifugation, soluble 12-mer arrays were quantified and plotted (middle). Shown are mean values +/- standard deviation, n = 3. For statistical analysis, the area under the curve was calculated and analyzed (right); shown are mean values with error bars representing 95% confidence intervals, n = 3, one-way ANOVA with Tukey's multiple comparisons test with $\alpha = 0.05$, 95% confidence interval, exact p values with $p \leq 0.05$ are indicated, ns (not significant) $p > 0.05$.

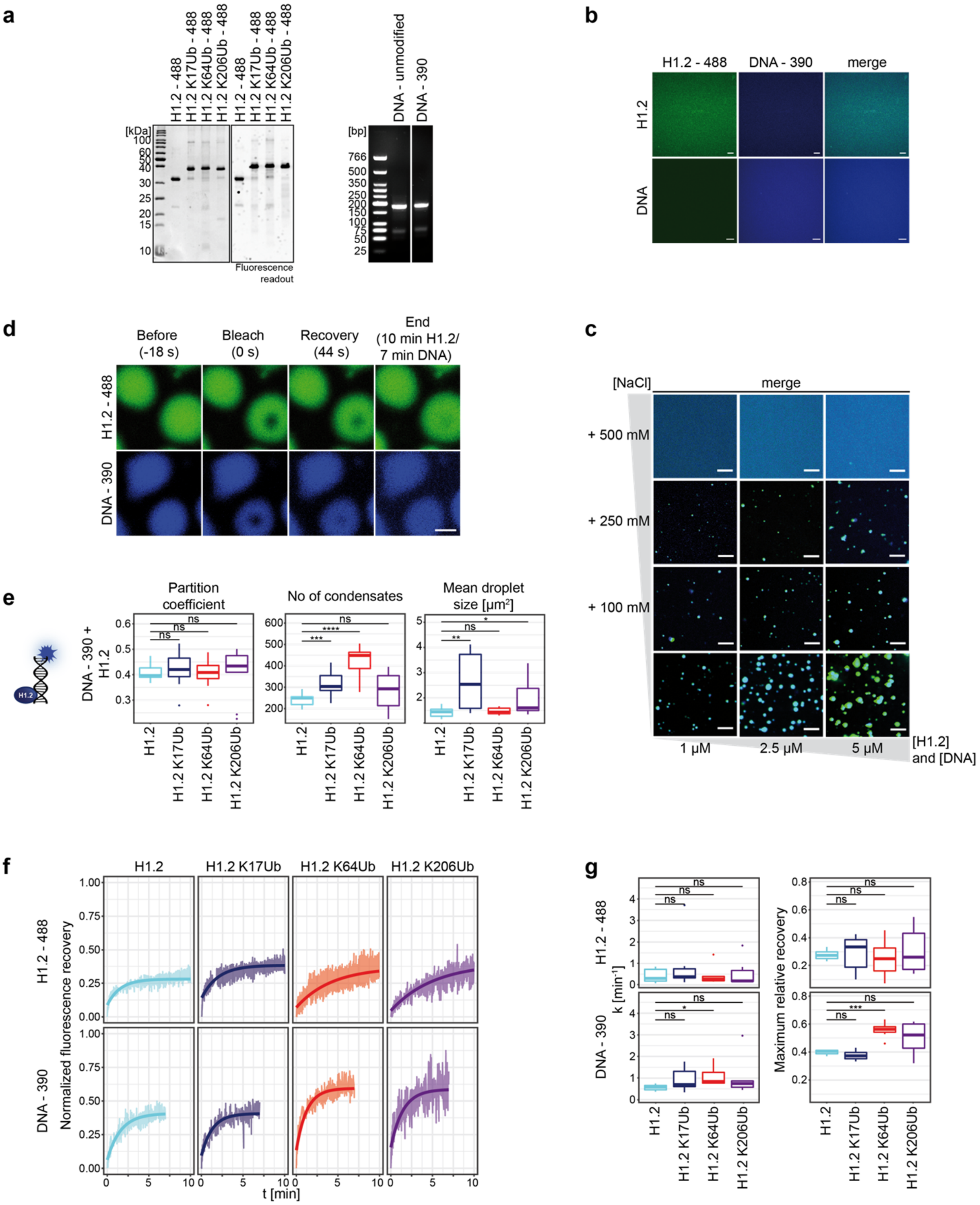

Supplementary Fig. 9: Phase separation of histones and DNA.

a Generation of histones labeled with Alexa Fluor 488 and DNA labeled with Atto 390 fluorescent dye. Labeled and unlabeled histones were mixed, separated by SDS-PAGE and visualized by Coomassie blue staining (left) and fluorescence readout (middle). 601 DNA was amplified and labeled by PCR and mixed with unlabeled DNA. DNA was separated by agarose gel electrophoresis and visualized by ethidium bromide staining (right). **b** Fluorescence microscopic images showing no droplet formation of pure labeled H1.2 (top) and pure labeled DNA (bottom). Scale bar 10 µm. **c** Phase diagram showing droplet formation using increasing concentrations of labeled H1.2 and DNA in 1x PBS with increasing NaCl concentrations. Merged images are shown; scale bar 10 µm. **d** Representative images of a time series of FRAP

analysis showing recovery of the bleached section over time. H1.2 was bleached with a laser at 488 nm (top) and DNA was bleached at 405 nm (bottom). Scale bar 2 µm. **e** Characterization of the DNA within H1.2- and H1.2 KxUb-DNA condensates. Shown are the partition coefficient (left), the number of droplets (middle) and mean droplet size (right) 20 min after mixing. Data were extracted from the images in the DNA (blue) channel and shown as boxplots where the midline represents the medians, the upper and lower bounds the interquartile ranges and the whiskers extend to 1.5 times the interquartile range, two-sample t-test, $n = 4$, exact p values with $p \leq 0.05$ are indicated, ns $p > 0.05$. **f** FRAP analysis of histone-DNA condensates. Depicted are representative fluorescence recovery curves of histones (top) and DNA (bottom). Recovery over several minutes for histones and DNA suggests a strong binding of DNA to H1.2/H1.2 KxUbs within the condensates. **g** Rate constant *k* and maximum relative recovery characterizing fluorescence recovery behavior are shown as boxplots as defined above, two-sample t-test; $n = 6$; exact p values with $p \leq 0.05$ are indicated, ns $p > 0.05$.

Supplementary Tables

Supplementary Table 1. Modification sites of endogenous H1.2.
Detected modifications of endogenous H1.2 after IP-enrichment are indicated within the identified peptide sequence: [-M] loss of initial methionine, (Ox) oxidation of methionine, (Ac) acetylation of lysine or protein N-terminus, (GG) or (LRGG) ubiquitylation, (Ph) phosphorylation, (Me) methylation. Only PTM-containing peptides are shown.

Peptide sequence	Precursor m/z [Da]	Char-ge	MH+(ex) [Da]	MH+(calc) [Da]	∆M [ppm]
**M**(Ox)SETAPAAPAAAPPAE**K**(GG)	870.41443	2+	1739.82158	1739.82688	-3.04
**M**(Ox)**S**(Ph)ETAPAAPAAAPPAE**K**(LRGG)	696.99860	3+	2088.98124	2088.7838	1.37
[-M]SETAPAAPAAAPPAEK	739.87659	2+	1478.74590	1478.74855	-1.79
[-M](Ac)-**S**ETAPAAPAAAPPAEK	760.88397	2+	1520.76067	1520.75911	1.02
[-M]**S**(Ph)ETAPAAPAAAPPAEK	520.23877	3+	1558.70176	1558.71488	-8.42
[-M]SE**T**(Ph)APAAPAAAPPAEK	779.85529	2+	1558.70329	1558.71488	-7.43
[-M](Ac)-**S**E**T**(Ph)APAAPAAAPPAEK	800.86456	2+	1600.72185	1600.72544	-2.25
[-M](Ac)-**S**ETAPAAPAAAPPAE**K**(Ac)	781.88147	2+	1562.75566	1562.76968	-8.97
MSETAPAAPAAAPPAEKAPV**K**(Ac)	683.02234	3+	2047.05246	2047.05285	-0.19
[-M](Ac)-**S**ETAPAAPAAAPPAEKAPVK	958.50702	2+	1916.00676	1916.01237	-2.93
[-M](Ac)-**S**ETAPAAPAAAPPAE**K**(Ac)APVK	979.51355	2+	1958.01982	1958.02293	-1.59
SGV**S**(Ph)LAALK**K**(GG)ALAAAGYDVE**K**(Ac)	1149.59167	2+	2298.17607	2298.17410	0.86
**K**(Ac)ALAAAGYDVEK	639.33850	2+	1277.66973	1277.67359	-3.03
ALAAAGYDVE**K**(Me)	561.29419	2+	1121.58110	1121.58372	-2.33
SLVS**K**(Ac)GTLVQT**K**(LRGG)GTGASGSF**K**(LRGG)	477.76913	6+	2861.57842	2861.59557	-5.99
G**T**(Ph)LVQT**K**(LRGG)GTGASGSF**K**(Ac)LNK	800.42303	3+	2399.25455	2399.24425	4.29
GTLVQT**K**(Ac)GTGASGSFK	790.91498	2+	1580.82268	158.82786	-3.28
**K**(Me)AGGT**K**(Ac)P**K**(Ac)	442.76260	2+	884.51793	884.51999	-2.33
AAKSAAKAVKPKAA**K**(Ac)P**K**(Ac)	438.02280	4+	1749.06936	1749.07452	-2.95

Supplementary Table 2. Oligonucleotides used for cloning and labeling of DNA.
Primers for site-directed mutagenesis were phosphorylated (Ph) at the 5’ end or modified with Atto-390 for fluorescence labeling.

Oligonucleotide	Sequence
H1.2 StrepII fwd	5′-GATCCATATGTGGAGCCACCCGCAGTTCGAAAAGGCTGCGAGCGAAACC GCACCGGCAGCACCTGCTGCA-3′
H1.2 StrepII rev	5′-AAGCTTGGATCCTTATTAGTGGTGATGGTGATGATG-3′
H1.2 His_6 fwd	5′-GGAGATATACATATGAGCGAAACCGCACCGGCAGCAC-3′
H1.2 His_6 rev	5′-AAGCTTGGATCCTTATTAGTGGTGATGGTGATGATGTTTTTTTTTCGGT GCCGCTTTCTTCGG-3′
H1.2 K17TAG fwd	5′-TAGGCACCGGTTAAAAAAAAAGCAGCC-3′
H1.2 K17TAG rev	5′-(Ph)-TTCTGCCGGAGGTGCTGCAGC-3′
H1.2 K64TAG fwd	5′-TAGGCACTGGCAGCAGCAGGTTATG-3′
H1.2 K64TAG rev	5′-(Ph)-TTTCAGTGCTGCCAGGCTAACAC-3′
H1.2 K206TAG fwd	5′-TAGAAAGCGGCACCGAAAAAAAAACATC-3′
H1.2 K206TAG rev	5′-(Ph)-CGGTTTAACCACTTTCGGTTTAGCG-3′
Ub StrepII fwd	5′-GATCCATATGTGGAGCCACCCGCAGTTCGAAAAGGCTGCGCAGATCTTC GTCAAGAC-3′
Ub StrepII rev	5′-GATCGGATCCTTATTACCCACCTCTGAGAC-3′
SIRT1 fwd	5′-CTACATATGGCGGACGAGGCGGCGG-3′
SIRT1 rev	5′-GTGCTCGAGTCCTCCGCGGCCGCTTGATTTG-3′
601 fwd	5′-CTATACGCGGCCGCCCTGG-3′
601 rev	5′-ATTCGGATCCACATGCACAGGATG-3′
601-Atto-390 rev	5′-ATTO390-(Ph)-ATTCGGATCCACATGCACAGGATG-3′
12-mer array fwd	5′-TAAGCAGAATTCGATATCACTAGTTCGGACCCTATACGCGG-3′
12-mer array rev	5′-ATTCGTAAGCTTGATATCGCTAGCGTATTAATTAATATGTATTCGGATC CAC-3′

Supplementary References

1. Bleher, R. & Martin, R. Nucleo-cytoplasmic translocation of histone H1 during the HeLa cell cycle. *Chromosoma* **108**, 308–316 (1999).

2. Green, A., Lonn, A., Peterson, K.H., Ollinger, K. & Rundquist, I. Translocation of histone H1 subtypes between chromatin and cytoplasm during mitosis in normal human fibroblasts. *Cytom. Part A* **77A**, 478–484 (2010).

3. Konishi, A. et al. Involvement of histone H1.2 in apoptosis induced by DNA double-strand breaks. *Cell* **114**, 673–688 (2003).
